# Response to ‘Body mass index and the risk of rheumatoid arthritis: a systematic review and dose–response meta-analysis’

**DOI:** 10.1186/s13075-015-0744-9

**Published:** 2015-08-19

**Authors:** Yang Liu, Cheryl Barnabe

**Affiliations:** Cumming School of Medicine, 3330 Hospital Dr. NW, Calgary, AB T2N 4N1 Canada

We address the recent paper by Qin *et al*. [1] evaluating the association between obesity and the development of rheumatoid arthritis (RA). They identified 11 cohort and case–control studies for meta-analysis, and estimated that obese individuals relative to overweight and normal weight individuals have an increased risk of RA (relative risk 1.25, 95 % confidence interval (CI) 1.07 to 1.45). We felt it important to complement their manuscript with a narrative description of results from studies not included in their synthesis, but which we have identified in our own research to be relevant to this discussion.

First, we highlight the studies that were not included in the Qin review but that also did not overlap with other cohorts used in the meta-analysis. Hemminki *et al*. [2] estimated a small but significant increase in RA risk in patients previously hospitalized for obesity relative to those who had not been, with a standardized incidence ratio of 1.37 (95 % CI 1.08 to 1.70). However, in two large cohort studies by Bartfai *et al*. [3] of an American Kaiser Permanente cohort, and by Vessey *et al*. [4] of a contraception study, no association between obesity and RA risk was found.

Associations between obesity and RA development have now also been studied in patients at high risk for progression to RA. van der Helm-van Mil *et al.* [5] investigated progression to RA in an undifferentiated arthritis cohort, and there was no evidence of an association with obesity. In a cohort of persons with arthralgias and/or a family history of RA, and with the presence of RA-specific anti-cyclic citrullinated peptide autoantibodies in their serum, de Hair *et al*. [6] found being overweight, independent of smoking, was associated with RA development (hazards ratio 5.6, 95 % CI 1.3 to 25.0, *P* = 0.023). Obesity was not studied per se.

These findings highlight the importance of a better understanding of the factors contributing to RA development, in particular any modifiable risk factors that we might address to reduce risk in various populations. We feel there is a signal that obesity is an important contributor to RA risk, but that further study is still merited.

## Abbreviations

CI: confidence interval
RA: rheumatoid arthritis
